# Use of Stent Retriever Thrombectomy Combined With Local Urokinase Thrombolysis for Deep Cerebral Vein Thrombosis: A Case Report

**DOI:** 10.3389/fstro.2022.901694

**Published:** 2022-07-14

**Authors:** Qunli Xu, Yigang Chen, Xu Zheng, Xing Jin, Jinhua Zhang

**Affiliations:** ^1^Zhejiang University, Hangzhou, China; ^2^Sir Run Run Shaw Hospital, Hangzhou, China

**Keywords:** case report, cerebral venous thrombosis (CVT), vein of Galen (VOG), internal cerebral vein thrombosis, basal vein of Rosenthal, local thrombolysis, mechanical thrombectomy (MT), endovascular treatment (EVT)

## Abstract

**Background:**

Deep cerebral vein (DCV) thrombosis is a rare type of cerebrovascular disorder and usually has an unfavorable prognosis. Although endovascular treatment measures for venous sinus thrombosis are well-described and evidenced, relevant reports on DCV thrombosis are presently lacking.

**Case Report:**

In this report, we present the first case of the successful use of stent retriever thrombectomy combined with local urokinase thrombolysis for DCV thrombosis and have described the detailed procedure.

**Conclusion:**

The use of stent retriever thrombectomy combined with local thrombolysis was technically feasible and effective for DCV thrombosis. The key to the successful restoration of DCV outflow was to recanalize the occluded vein of Galen.

## Introduction

Cerebral venous thrombosis (CVT) is a rare type of cerebrovascular disorder causing 0.5% of stroke cases (Sader et al., 2018). Approximately 10.6% of CVT patients were severely paralyzed or dead at the end of follow-up in a previous report (Ferro et al., 2004). Approximately 10.9% of patients with CVT had thrombosis of deep cerebral veins (DCVs; Ferro et al., 2004; Kiliç and Akakin, 2008), mainly the vein of Galen (VOG) and the veins close to VOG, including internal cerebral vein (ICV) and basal vein of Rosenthal (BVR). Pregnancy, puerperium, oral contraceptive use, and infections were the most common predisposing factors for thrombosis of DCVs (Sagduyu et al., 2006). Deep cerebral veins drain the central structures of the cerebral hemispheres, basal ganglia, corpus callosum, pineal region of the limbic system, and thalami (Kiliç and Akakin, 2008). Therefore, DCV thrombosis can cause isolated mental status disorder, which makes the diagnosis challenging in the early stage of the disorder. Despite first-line anticoagulation therapies, DCV thrombosis can lead to three times the risk of death or dependency compared to CVT at other sites, often making the treatment futile (Ferro et al., 2004).

Endovascular treatment (EVT) is considered in patients with severe CVT after adequate anticoagulation treatment failure and/or when there is a need to prevent or treat brain herniation (Fan et al., 2020), although evidence from randomized clinical trials is lacking (Ferro et al., 2017; Coutinho et al., 2020). Consensus recommendations for the management of venous sinus thrombosis are relatively clear and substantiated (Saposnik et al., 2011; Ferro et al., 2017; Fan et al., 2020; Yeo et al., 2020), while management strategies for DCV thrombosis remain uncertain due to the lack of relevant reports.

In this report, we present a case of the successful use of stent retriever thrombectomy combined with local urokinase thrombolysis for thrombosis of VOG, ICV, and BVR.

## Case Presentation

**Figure 1:**
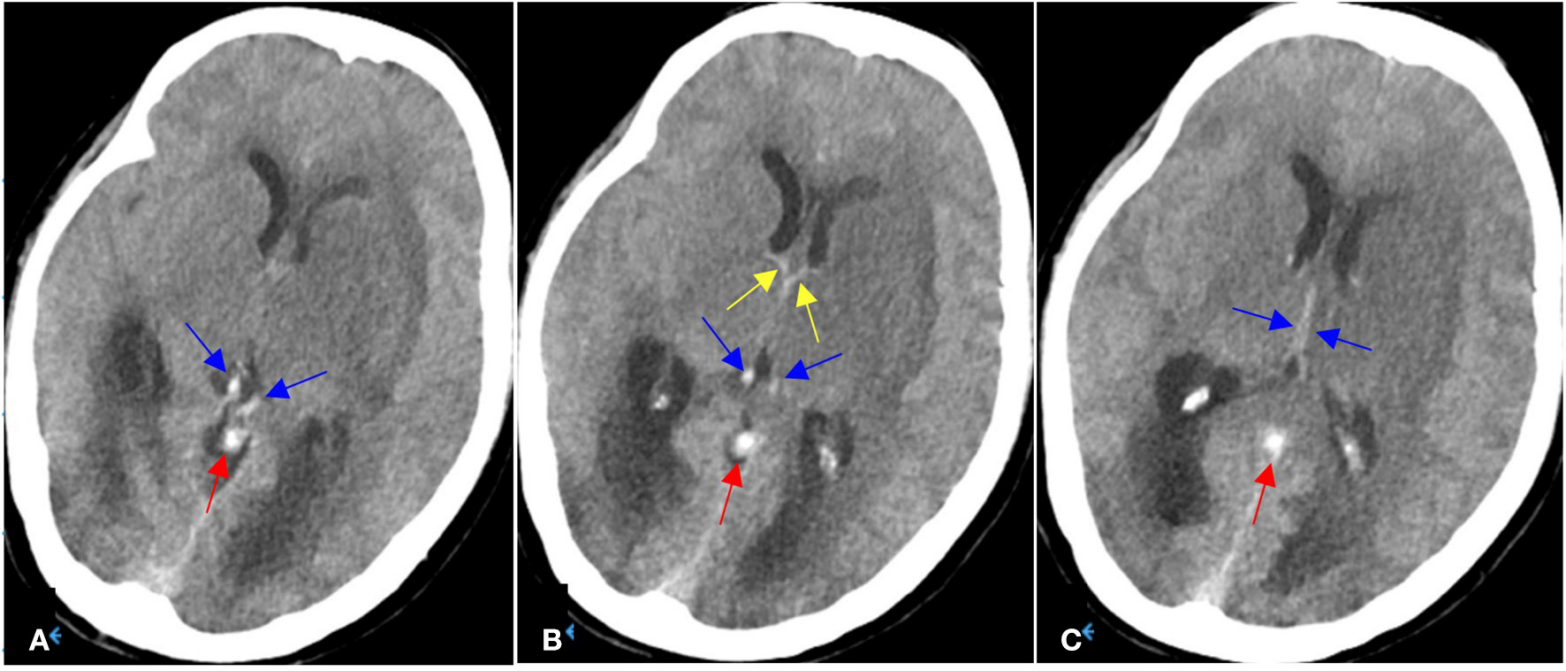
**(A–C)** On Aug 8, 2021, emergent head NCCT demonstrated hypodensities and edema within bilateral thalami and basal ganglia (left side being more severe) with mild obstructive hydrocephalus, as well as hyperdense thalamostriate vein (yellow arrows), internal cerebral veins (blue arrows) and vein of Galen (red arrows).

**Figure 2:**
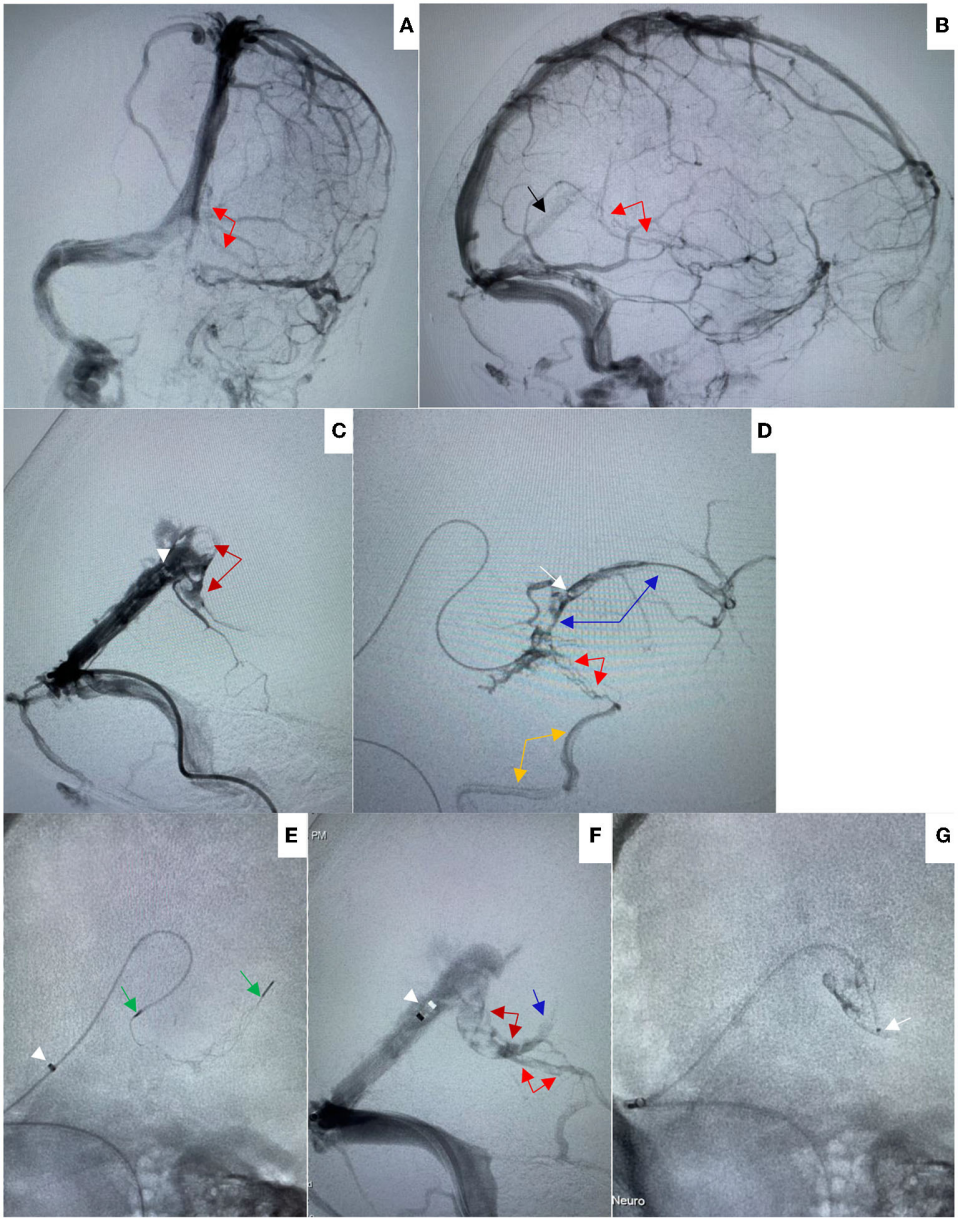
**(A,B)** On Aug 8, 2021, pre-procedural left internal carotid artery (ICA) angiogram in anteroposterior (AP) **(A)** and lateral **(B)** views showed good opacification of straight sinus (black arrow) without opacification of internal cerebral vein and vein of Gallen, and with poor opacification of basal vein of Rosenthal (red arrows). **(C)** Venography through Catalyst 6 (white arrowhead) showed filling defect in the vein of Galen (dark red arrows) suggesting thrombosis. **(D)** Venography through microcatheter (white arrow) showed filling defect in the internal cerebral vein (blue arrows) and basal vein of Rosenthal suggesting thrombosis (red arrows). The basal vein was seen to drain *via* the inferior temporal vein (orange arrow) toward the transverse sinus. **(E)** A 4 × 20 mm Trevo stent retriever was temporarily deployed in the vein of Galen and proximal internal cerebral vein through the microcatheter (white arrowhead indicated the tip of Catalyst 6, green arrows indicated the distal and proximal markers of the stent retriever). **(F)** After a single thrombectomy pass, venography through Catalyst 6 showed the blood flow was significantly improved, the filling defects were significantly reduced in the vein of Galen (dark red arrows), basal vein of Rosenthal (red arrows), and proximal internal cerebral vein (blue arrow). **(G)** The microcatheter was preserved in the vein of Galen.

**Figure 3:**
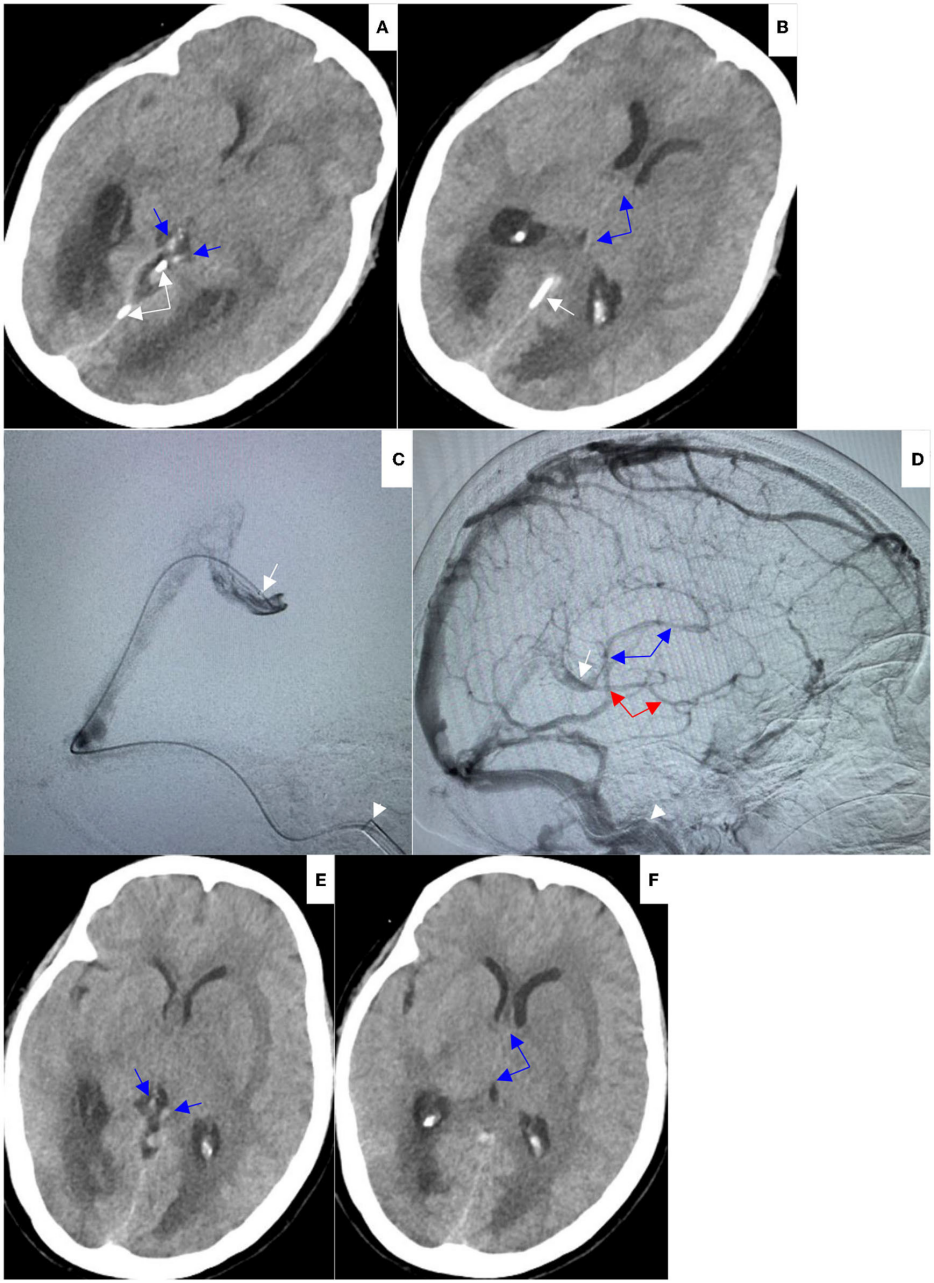
**(A,B)** On Aug 9, 2021, repeat head NCCT demonstrated hyperdensities in internal cerebral veins (blue arrows) and vein of Galen were decreased (white arrows indicated the microcatheter). **(C,D)** On Aug 9, 2021, repeat venography through preserved microcatheter in lateral view **(C)** showed excellent recanalization of vein of Galen (white arrowhead indicated the tip of Mach 1); repeat left internal carotid artery angiogram in lateral view **(D)** showed good opacification of internal cerebral vein (blue arrows) and basal vein of Rosenthal (red arrows). **(E,F)** On Aug 10, 2021, repeat head NCCT showed hyperdensities in internal cerebral veins (blue arrows) were further decreased, hypodensities and edema within bilateral thalami and basal ganglia were significantly improved, and the obstructive hydrocephalus was relieved.

A 54-year-old Chinese female was brought to the emergency department due to a coma vigil. The patient had started experiencing headaches 15 days earlier and had not taken any remedial measures. Three days before admission, she suddenly had unconsciousness with incontinence and was immediately taken to the local hospital. Subsequently, she was suspected of having viral encephalitis, autoimmune encephalitis, or Wernicke encephalopathy and received antiviral therapy, intravenous steroids, and vitamin B1 supplement. However, there was no improvement in her condition. In our hospital, an emergent head non-contrast computed tomography (NCCT) scan revealed edematous lesions within bilateral thalami and basal ganglia with mild obstructive hydrocephalus, as well as hyperdensities of thalamostriate vein, ICV, and VOG, indicating DCV thrombosis (Figures 1A–C). Therefore, an emergent EVT was performed under general anesthesia. A bolus dose of heparin (50 U/kg), followed by 1,000 U/h, was intravenously administered during the procedure. A pre-procedural angiogram was performed through the internal carotid artery to confirm occlusion of VOG, ICV, and BVR with a patent straight sinus (Figures 2A,B). An 8F guide catheter (Mach 1; Boston Scientific Corp., Natick, MA, USA) with a 5F MPA catheter using a coaxial technique was navigated into the distal segment of the right internal jugular bulb through the right femoral vein. Under the guidance of a microcatheter (Trevo Pro-18; Stryker, Fremont, CA, USA), over a 0.014-inch microwire (Synchro-2, Stryker), an AXS catalyst 6 (Stryker) was successfully advanced into the straight sinus but failed to enter VOG. Angiogram through catalyst 6 (white arrow) confirmed thrombosis in the VOG (Figure 2C). Then, the microcatheter was advanced into the left ICV, and venography confirmed thrombosis in ICV and BVR (Figure 2D). Subsequently, a 4 × 20 mm Trevo stent retriever (Stryker) was temporarily positioned in the proximal ICV and VOG through the microcatheter (Figure 2E). When the thrombus was retrieved by the stent retriever, the negative pressure within the catalyst 6 was retained with the use of a 50 ml vacuum syringe. After a single thrombectomy pass, the blood flow was significantly improved in the DCVs (Figure 2F). Then, the microcatheter was introduced into the VOG again and positioned there (Figure 2G). After that, catalyst 6 was retrieved. A bolus dose of 50,000 U of urokinase, followed by continuous urokinase, was administered into the VOG through the microcatheter (500,000 U of urokinase and normal saline were mixed to prepare a 48 ml solution, which was continuously pumped through the infusion pump at a rate of 4 ml/h). Systemic heparinization was performed by continuous administration of heparin through the guide catheter to maintain an activated partial thromboplastin time (pat) of 1.5–2.0 times the normal value. Awaken from general anesthesia, the patient was conscious and alert, and the tracheal intubation was removed within 2 days. Repeated head NCCT showed that edematous lesions within bilateral thalami and basal ganglia were gradually regressed (Figures 3A,B,E,F). Repeated venography and angiogram in 15 h demonstrated the excellent recanalization of VOG, left ICV, and BVR (Figures 3C,D). Then, the administration of urokinase through the microcatheter and heparin through the guide catheter was discontinued. Anticoagulation therapy was subsequently started with low molecular weight heparin calcium 6,000 IU twice a day for 1 week, followed by long-term oral rivaroxaban 20 mg once a day for 6 months. The results of extensive analyses for the prothrombotic state, including anti-nuclear antibody profile, antineutrophil cytoplasmic antibody profile, antiphospholipid antibody test, and screening for protein C, protein S, or antithrombin III deficiencies, were negative. The homocysteine level was 15.5 μmol/L.

## Follow-up Imaging and Clinical Outcome

**Figure 4:**
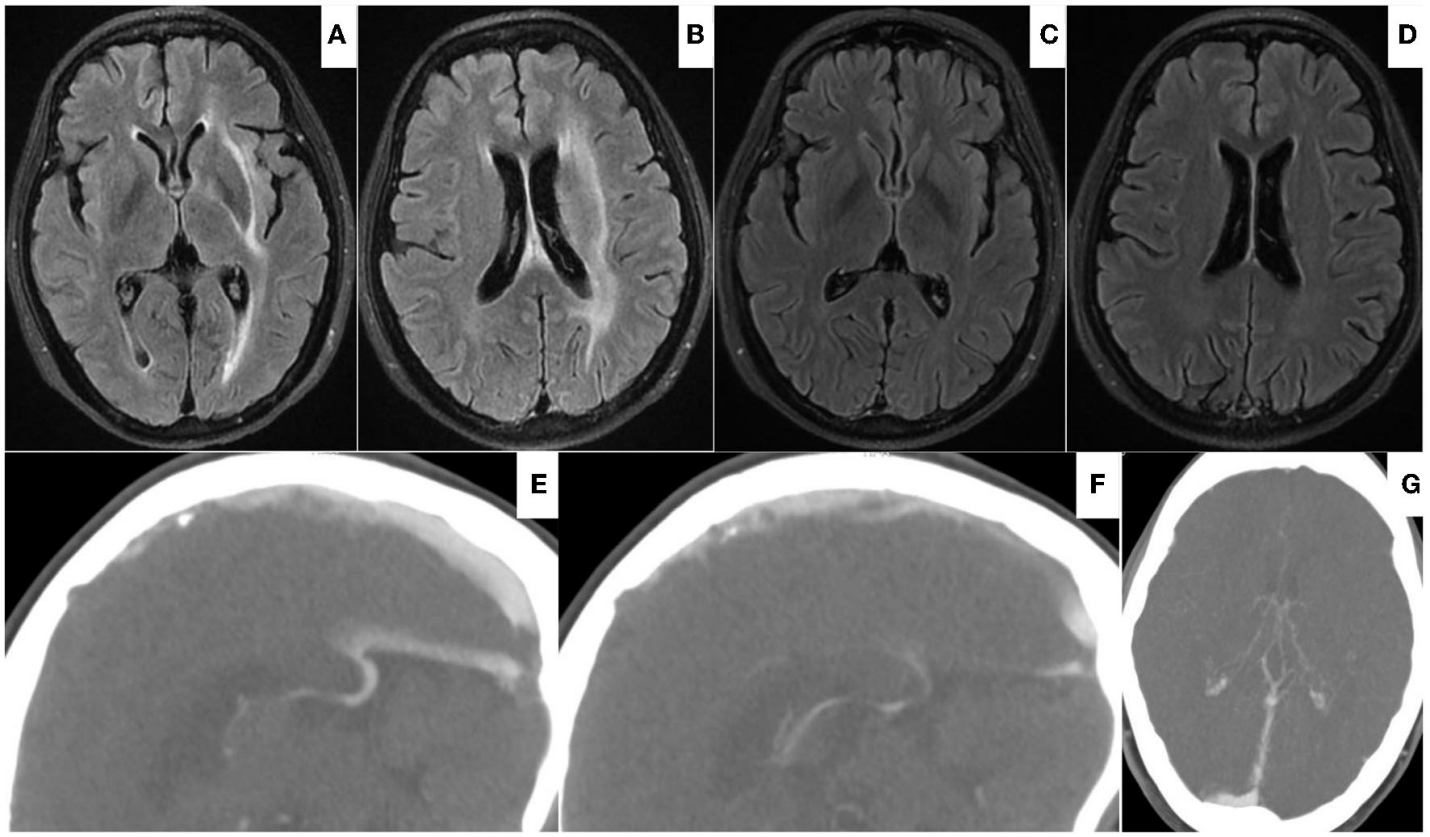
**(A,B)** On Aug 19, 2021, head FLAIR-MRI demonstrated edematous lesions within bilateral thalami and basal ganglia significantly regressed with residual hyperdense at left internal capsule, external capsule, and periventricular area. **(C–F)** On Oct 22, 2021, follow-up FLAIR-MRI showed no residual parenchymal defects **(C,D)**; CTV showed patency of vein of Galen and internal cerebral vein **(E–G)**.

Ten weeks after the EVT, MRI, and CT venography revealed the disappearance of edema from the bilateral thalami and basal ganglia with patency of DCVs (Figure 4). Repeated testing for protein C, protein S, and antithrombin III deficiency showed negative results, but the D-dimer level remained high (2.03 μg/ml; reference level: 0.5 μg/ml) 1 month after the discontinuation of anticoagulation therapy. Upon rechecking, the homocysteine level was normal. The etiology of this case was not yet clear, and follow-up of the patient was ongoing when drafting this report. The modified Rankins scale score (mRS) was 0 at the 90-day and 180-day follow-up.

## Discussion

In the present case, an effective imaging technique was employed in the diagnosis, and good clinical outcomes were achieved by emergent stent retriever thrombectomy combined with local urokinase thrombolysis therapy. Patients with DCV thrombosis whose anticoagulation therapies are ineffective or who experience impaired consciousness usually have an unfavorable prognosis. The lack of evidence for other effective treatments makes clinical intervention unsuccessful. Endovascular treatment measures that are commonly used in severe thrombosis of superior sagittal, transverse, and sigmoid sinuses, such as mechanical thrombectomy, local application of alteplase or urokinase, or a combination of both strategies, have recently been reported to be effective for straight sinus thrombosis (Matsuda et al., 2017; Taniguchi et al., 2017; Coutinho et al., 2020; Sundar et al., 2021; Yang et al., 2021). However, reports of EVT for classic DCV thrombosis are still lacking. To the best of our knowledge, the present case was the first relevant report on managing DCV thrombosis.

The present case demonstrated the use of a stent retriever combined with local thrombolysis in greatly aiding the removal of the thrombi located in DCVs. As the core of DCVs, VOG mainly receives blood flow from the bilateral ICVs and BVRs (Ferro et al., 2004; Kiliç and Akakin, 2008). Anatomically, the adult VOG is a collector of multiple drainages. It can distribute its drainage anteriorly to the cavernous sinus *via* the BVR, laterally toward the inferior temporal vein, and posteriorly toward the straight sinus. Because of these anastomoses, only simultaneous obstructions of the VOG and BVRs will impede deep venous outflow (Krings et al., 2015). Therefore, the key point of successful restoration of DCVs' outflow is to recanalize the occluded VOG. This was the reason why we performed both thrombectomy and local thrombolysis in the VOG.

In the present case, a 0.021-inch microcatheter was successfully introduced into the ICV, while catalyst 6 could not follow even after the stent retriever was anchored in the VOG. It may have been due to the steep and sharp angle at the junction of the VOG and straight sinus. Besides, studies suggest that 42% of normal DCVs have stenosis (width and thickness of ≤2 mm) or an impression of the arachnoid granulation (articular shape) at the junction (Hou et al., 2021). Furthermore, we should bear in mind that the wall of the vein is far thinner than the arteries, and DCVs are not fixed but float in the subarachnoid space. Straightening or violent manipulation of the vein during the intervention may lead to perforation or rupture of DCVs, which can have fatal bleeding consequences. Therefore, the microguidewire and microcatheter must be manipulated gently while passing through the junction and must not suddenly increase tension to pass when encountering resistance. Because of the risks of vascular perforation and rupture, the number of stent retriever thrombectomy operations should be as few as possible, and the operation is not required once the VOG has partially been recanalized. During the interventional procedure, the role of the stent retriever is to reduce the thrombus load to restore the venous outflow channel and simultaneously rupture and loosen the thrombus to increase the contact area between urokinase and the thrombus. Removal of remaining thrombus in DCVs is left to the local thrombolysis. Microcatheter local thrombolysis has relatively low requirements of materials and techniques, is simple and fast to operate, and has little risk of vascular intima injury. Therefore, it should be a more reasonable intervention strategy. Moreover, when the aspiration catheter fails to deliver to VOG, the stent retriever could be an option for thrombectomy for DCV thrombosis. Since over 40% of normal DCVs have stenosis variation (Hou et al., 2021), a 4 mm nominal diameter stent tailored for large intracranial arteries is obviously not the best choice. In the future, the introduction of a smaller stent retriever tailored for intracranial veins may enable the neurointerventionalist to safely treat these patients.

As the lesions in the left brain are much more serious than those in the right brain, we chose to operate in the left ICV. Meanwhile, the Trevo retriever was positioned in the ICV instead of BVR because the BVR is not as constant as the ICV anatomically and may have discontinuous variation (Rouchaud et al., 2016; Hou et al., 2021).

This was a typical case of DCV thrombosis with delayed treatment due to misdiagnosis. Although NCCT diagnosis of venous sinus thrombosis had a low sensitivity of about 30%, the sensitivity, and specificity of the attenuated vein sign-on NCCT scan for the diagnosis of DCV thrombosis were 100 and 99.4%, respectively (Linn et al., 2009). As it is fast and widely available, head NCCT is still used as the first-line diagnostic approach in the emergency setting in most Chinese institutions. Therefore, in a patient with unilateral or bilateral thalamic edema on an NCCT scan, clinicians should pay attention to direct signs (the presence of hyperattenuating veins) of DCV thrombosis to avoid misdiagnosis.

## Take-Home Messages

- CVT that occurs only in the deep cerebral veins (DCVs) without involving the dural sinuses is unusual. Hence, it is easy to misdiagnose without catheter angiography. Clinicians should pay close attention to the subtle features on NCCT scans (hyperdense ICVs with thalamic edema).
- Use of stent retriever thrombectomy combined with local thrombolysis is technically feasible and effective for DCV thrombosis. Considering the anatomical characteristics, mechanical thrombectomy does not aim to achieve a perfect angiographically normal vein but to reduce the burden of the clot to allow thrombolysis.
- The key to the successful restoration of DCVs' outflow is to recanalize the occluded VOG.
- If the patient with DCV thrombosis develops unconsciousness from severe thalamic edema, endovascular treatment could be considered.

## Data Availability Statement

The original contributions presented in the study are included in the article/supplementary material, further inquiries can be directed to the corresponding author.

## Ethics Statement

The studies involving human participants were reviewed and approved by Sir Run Run Shaw Hospital Ethics Committee School of Medicine, Zhejiang University. The patient provided her written informed consent to participate in this study. Written informed consent was obtained from the individual(s) for the publication of any potentially identifiable images or data included in this article.

## Author Contributions

QX and JZ: study design, acquisition of data, image processing, image analysis, data analysis, and drafting the manuscript and revising it critically. YC, XZ, and XJ: data analysis and drafting the manuscript and revising it critically. All authors contributed to the article and approved the submitted version.

## Conflict of Interest

The authors declare that the research was conducted in the absence of any commercial or financial relationships that could be construed as a potential conflict of interest.

## Publisher's Note

All claims expressed in this article are solely those of the authors and do not necessarily represent those of their affiliated organizations, or those of the publisher, the editors and the reviewers. Any product that may be evaluated in this article, or claim that may be made by its manufacturer, is not guaranteed or endorsed by the publisher.
